# Investigation of the Hydrogen Sulfide Signaling Pathway in Schwann Cells during Peripheral Nerve Degeneration: Multi-Omics Approaches

**DOI:** 10.3390/antiox11081606

**Published:** 2022-08-19

**Authors:** Yoo Lim Chun, Won-Joon Eom, Jun Hyung Lee, Thy N. C. Nguyen, Ki-Hoon Park, Hyung-Joo Chung, Han Seo, Youngbuhm Huh, Sang Hoon Kim, Seung Geun Yeo, Wonseok Park, Geul Bang, Jin Young Kim, Min-Sik Kim, Na Young Jeong, Junyang Jung

**Affiliations:** 1 Department of Anatomy and Neurobiology, College of Medicine, Kyung Hee University, Dongdaemun-gu, Seoul 02447, Korea; 2 Department of Biomedical Science, Graduation School, Kyung Hee University, Dongdaemun-gu, Seoul 02447, Korea; 3 Department of Anatomy and Cell Biology, College of Medicine, Dong-A University, Seo-gu, Busan 49201, Korea; 4 Department of New Biology, Daegu Gyeongbuk Institute of Science and Technology (DGIST), Dalseong-gu, Daegu 42988, Korea; 5 Department of Anesthesiology and Pain Medicine, College of Medicine, Kosin University, Seo-gu, Busan 49267, Korea; 6 Department of Otorhinolaryngology-Head and Neck Surgery, College of Medicine, Kyung Hee University, Dongdaemun-gu, Seoul 02447, Korea; 7 Department of Orthopedic Surgery, Good Samsun Hospital, Sasang-gu, Busan, 47007, Korea; 8 Research Center for Bioconvergence Analysis, Korea Basic Science Institute, Ochang 28119, Korea

**Keywords:** Schwann cells, hydrogen sulfide, peripheral nerve degeneration, multi-omics, *N*-ethylmaleimide (NEM), oxidative stress

## Abstract

*N*-ethylmaleimide (NEM) inhibits peripheral nerve degeneration (PND) by targeting Schwann cells in a hydrogen sulfide (H_2_S)-pathway-dependent manner, but the underlying molecular and pharmacological mechanisms are unclear. We investigated the effect of NEM, an α,β-unsaturated carboxyl compound, on H_2_S signaling in *in vitro*- and *ex vivo*-dedifferentiated Schwann cells using global proteomics (LC-MS) and transcriptomics (whole-genome and small RNA-sequencing (RNA-seq)) methods. The multi-omics analyses identified several genes and proteins related to oxidative stress, such as *Sod1*, *Gnao1*, *Stx4*, *Hmox2*, *Srxn1*, and *Edn1*. The responses to oxidative stress were transcriptionally regulated by several transcription factors, such as *Atf3*, *Fos*, *Rela*, and *Smad2*. In a functional enrichment analysis, cell cycle, oxidative stress, and lipid/cholesterol metabolism were enriched, implicating H_2_S signaling in Schwann cell dedifferentiation, proliferation, and myelination. NEM-induced changes in the H_2_S signaling pathway affect oxidative stress, lipid metabolism, and the cell cycle in Schwann cells. Therefore, regulation of the H_2_S signaling pathway by NEM during PND could prevent Schwann cell demyelination, dedifferentiation, and proliferation.

## 1. Introduction

Schwann cells are unique neuroglial cells that form myelin sheaths around axons in the peripheral nervous system (PNS). Schwann cells also play important roles in peripheral nerve degeneration (PND). For example, during PND, Schwann cells dedifferentiate, crush the myelin sheath, and proliferate to promote nerve regeneration [1]. Unlike mechanical nerve injury, continuous systemic nerve damage such as hyperglycemia can lead to irreversible PND and ultimately cause peripheral neurodegenerative diseases such as diabetic peripheral neuropathy [2]. Malfunction or nonfunction of Schwann cells under hyperglycemia is an important cause of irreversible PND. Furthermore, because the pathological mechanism of peripheral neurodegenerative diseases is unknown, clinical treatment is limited to their symptoms.

Hydrogen sulfide (H_2_S) is an inorganic gaseous signaling molecule in cells [3]. H_2_S is endogenously generated, present in all organs, and diffuses intracellularly and intercellularly [3]. H_2_S is a mediator of several physiological and pathological events, such as synaptic transmission, inflammation, angiogenesis, vascular tone, apoptosis, cell cycle, and oxidative stress [4]. In the nervous system and cardiovascular system, H_2_S exerts cardioprotective and neuromodulatory effects, respectively [5]. In mammalian cells, endogenous biosynthesis of H_2_S requires organ-specific enzymes that generate H_2_S, starting from L-cysteine. Four such enzymes have been identified: cystathionine γ-lyase (CSE), cystathionine β-synthase (CBS), cysteine aminotransferase (CAT), and 3-mercaptopyruvate sulfotransferase (MST) [6]. In the central nervous system, CBS, rather than CSE, is the main H_2_S-producing enzyme, whereas CSE, and not CBS, is present in the PNS and regulates Schwann cell dynamics during PND [7,8]. Therefore, regulation of the expression of H_2_S-producing enzymes affects many biological events in living cells.

*N*-Ethylmaleimide (NEM) is an *N*-ethyl derivative of maleic acid. NEM inhibits substrate–enzyme combination and the catalytic reaction [9]. In the PNS, NEM maintains the properties of myelinated Schwann cells and prevents demyelination and proliferation of dedifferentiated Schwann cells [8]. NEM inhibits H_2_S-producing enzymes, thus regulating the expression of CSE and MST, and significantly affects H_2_S signaling in Schwann cells during PND [8]. However, the underlying molecular mechanisms are unclear. We performed a multi-omics analysis using in vitro and ex vivo PND models. The findings showed that the H_2_S signaling pathway was regulated at the transcriptional, post-transcriptional, and translational levels, and NEM acted as an antioxidant to regulate the response to oxidative stress during PND.

## 2. Materials and Methods

### 2.1. Animals

Five-week-old C57BL/6 male mice from Orientbioa^TM^ (Seongnam, Korea) were used in the study. Animals were housed under standard conditions with a 12 h light/dark cycle and provided with free access to water and feed. All animal experiments were performed in full compliance with the guidance of Korean Academy of Medical Science and with the approval of Kyung Hee University Committee of Animal Research [KHSASP-21-463].

### 2.2. Ex Vivo Sciatic Nerve Culture

Mouse sciatic nerve explant cultures were prepared as described previously [8]. The sciatic nerve was exposed after skin incision with a fine iris scissor (FST, Foster City, CA, USA) and blunt dissection of the overlying muscles. A tight ligation around the sciatic nerve was detached under a stereomicroscope in the phosphate-buffered saline (PBS). Each explanted sciatic nerve was divided into 3 or 4 pieces 3–4 mm in length. After cutting sciatic nerves, the nerves were cultured in Dulbecco’s modified Eagle’s medium (DMEM, Hyclone, Waltham, MA, USA) supplemented with 9% (*v/v*) fetal bovine serum (FBS, Hyclone, Waltham, MA, USA) and 1% (*v/v*) penicillin/streptomycin (Corning, New York, NY, USA). Sciatic nerves were cultured in 24-well dishes and incubated in a humidified atmosphere containing 5% of CO2 at 37 °C for one day.

**Figure 1:**
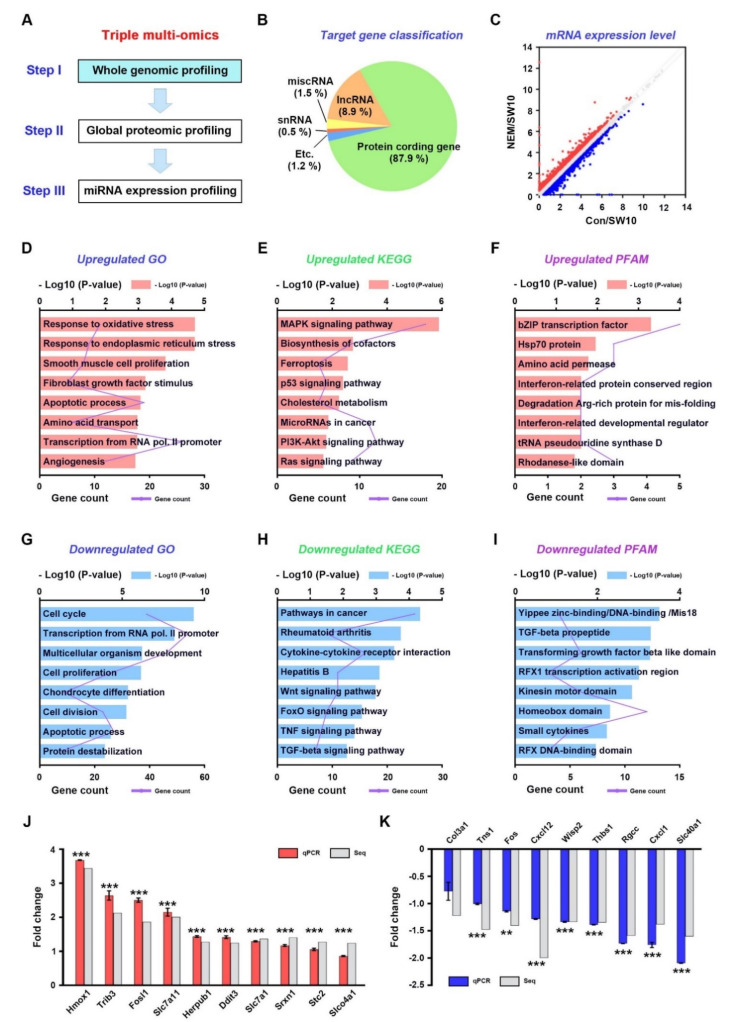
Whole genomic analysis with NEM as an inhibitor for hydrogen sulfide pathway using SW10 cells. (**A**) The first stage of a three-stage framework for integrating triple multi-omics data. The SW10 cell culture was performed for 5 h after NEM treatment. (**B**) The pie chart represents detected genes into five summarized transcript types. (**C**) A scatter plot of pairwise expressed genes. *X*-axis and *y*-axis present log2 value of normalized gene expression. Red, blue, and gray dots refer to upregulated, downregulated, and nonregulation genes. GO terms (**D**), KEGG pathways (**E**), and PFAM (**F**) enrichments of upregulated genes are shown. GO terms (**G**), KEGG pathways (**H**), and PFAM (**I**) enrichments of downregulated genes are shown. qPCR validation of the RNA-sequencing (Seq) in the upregulated (**J**) and downregulated (**K**) top 10 genes. Seq, RNA sequencing. Comparisons were performed using a Student’s *t*-test. ** *p* < 0.01 and *** *p* < 0.001.

### 2.3. Cell Culture and Chemicals

The mouse neuronal Schwann cell line SW10 cells were purchased from American Type Culture Collection (ATCC, Manassas, VA, USA) and cultured in DMEM added with 9% (*v*/*v*) FBS and 1% (*v*/*v*) penicillin/streptomycin at 37 ℃ with 5% CO_2_. *N*-Ethylmaleimide (Sigma-Aldrich, St. Louis, MO, USA) was dissolved in dimethyl sulfoxide (DMSO, Life science, Randnor, PA, USA) and treated in the culture at 10 μM concentration for 5 h.

### 2.4. Proteomic Sample Preparation

Proteins were extracted with a lysis buffer (50 mM Tris-HCl pH 8.0) by ultrasonication (Sonic Dismebrator 500, Thermo Fisher Scientific, Waltham, MC, USA) with 10% amplitude. Peptide samples were prepared as described previously [10]. After centrifugation, clear supernatant protein lysates were transferred to a new tube. Proteins were reduced with 10 mM DTT, alkylated using 50 mM IAA in dark for 30 min, and enzymatically digested with a sequencing-grade trypsin at 37 °C with a protein-to-enzyme ratio of 25 to 1 (*w*/*w*) overnight. After quenching with an acidic solution (1% formic acid), peptides were desalted using a C18 micro spin column (Harvard Apparatus, Cambridge, MC, USA) and eluates were dried using a speed vac prior to TMT labeling. Six-plex TMT kit was used to label six samples. A total of 100 μg of proteins were used according to a manufactural protocol.

### 2.5. Tandem Mass Tag (TMT)-Based Quantitative Proteomics

TMT-labeled peptides were combined prior to offline bRPLC fractionation. The linear gradient was performed using buffer A (10 mM TEAB in water) and buffer B (10 mM TEAB in 90% acetonitrile), and a total of 14 fractions were analyzed using an LC-MS/MS system consisting of an EASY nLC system (Thermo Fisher Scientific) and an Orbitrap Lumos mass spectrometer (Thermo Fisher Scientific) equipped with a nano-electrospray source (EASY-Spray Sources, Thermo Fisher Scientific). Peptides were first loaded onto a trap column (75 μm × 2 cm C18 precolumn, nanoViper, Acclaim PepMap100, Thermo Fisher Scientific) prior to separation on an analytical C18 column (75 μm × 50 cm PepMap RSLC, Thermo Fisher Scientific) at a flow rate of 300 nL/min. Mobile phases A and B were composed of 100% water containing 0.1% formic acid and 100% acetonitrile contained 0.1% formic acid, respectively. The LC gradient began with 5% B and was stayed at 5% B for 5 min, ramped to 30% B for 85 min, to 95% B for 10 min, and remained at 95% B over 5 min. Finally, it was ramped to 5% B for another 5 min. The column was re-equilibrated with 5% B for 5 min before the next run. The voltage of 1800 V was applied to produce an electrospray, and the Orbitrap mass spectrometer was operated in a data-dependent mode by switching MS1 and MS2 automatically. The MS data were acquired using the following parameters: Full scan MS1 spectra (400–1600 m/z) were acquired in the Orbitrap for a maximum ion injection time of 50 ms at a resolution of 120,000 and a standard mode automatic gain control (AGC) target. MS2 spectra were acquired in the Orbitrap mass analyzer at the resolution of 50,000, applying high energy collision dissociation (HCD) of 37.5% normalized collision energy and AGC target value of 5.0 × 10^4^ with a maximum ion injection time of 50 ms. Previously fragmented ions were excluded for 20 s.

### 2.6. Proteomic Data Analysis

Mass-spectrometry raw files were processed by MaxQuant (ver 2.1.0.0) [10] and tandem mass spectra were searched against mouse reference proteins downloaded from Uniprot database (21,985 entries; https://.uniprot.org/; accessed on 25 March 2022). Default search parameters were applied with carbamidomethylation on cysteine as a fixed modification and with methionine oxidation and protein *N*-terminal acetylation as variable modifications. TMT 6-plex was selected for reporter ion ms2. Mass tolerance for the first search and the main search were 20 ppm and 4.5 ppm, respectively. The peptide length of minimum 7 amino acids with maximum 2 missed cleavage sites by trypsin were required. False discovery rates (FDRs) were set at 1% for both peptides and proteins. Further analyses were processed in Perseus (ver 1.6.15.0) [11]. After the removal of reverse sequences and potential contaminants, reporter intensities were log2-transformed and quantile normalized for the data analysis. Clustered heatmaps and trend plots were illustrated by RStudio (ver 1.4.1106) with ComplexHeatmap and ggplot packages, respectively.

**Figure 2:**
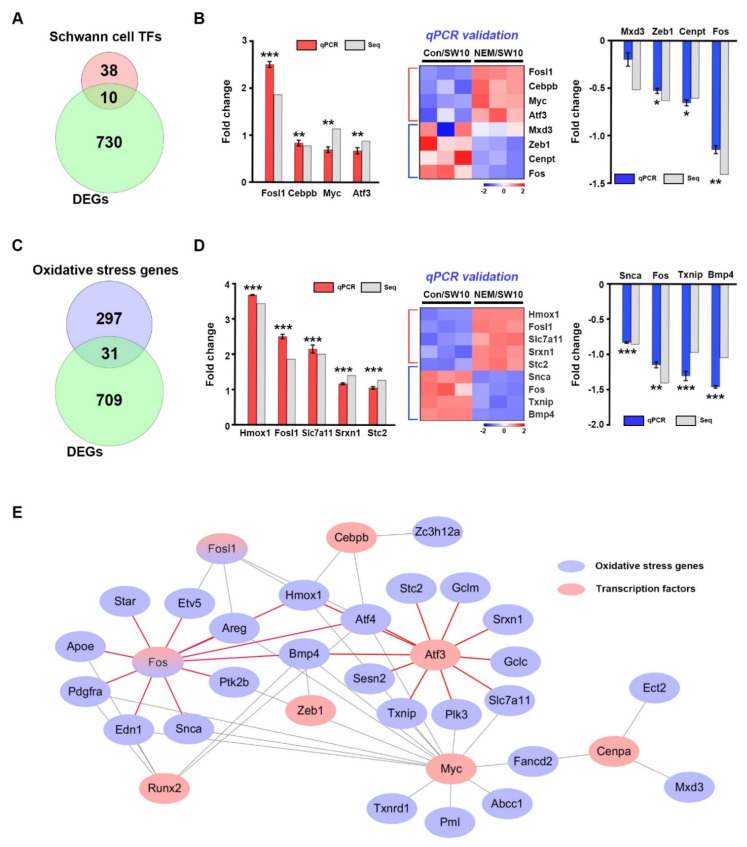
Interaction between oxidative stress genes and transcription factors at the transcriptomic level. (**A**) Venn diagram shows transcription factor (TF) genes involved in their in vivo expression among differentially expressed genes (DEGs). (**B**) Four upregulated and four downregulated transcription factor genes were validated by qPCR. Heatmap shows the relative expression levels of genes. Seq, RNA sequencing. (**C**) Venn diagram shows oxidative stress genes among differentially expressed genes (DEGs). (**D**) Validation of the expression of nine oxidative stress genes by qPCR showed increased expression of five genes and decreased expression of four genes. Heatmap shows the relative expression levels of genes. (**E**) Interconnection network between oxidative stress and transcription factor genes. The red and purple colors denote transcription factors and oxidative stress genes, respectively. Comparisons were performed using a Student’s *t*-test. * *p* < 0.05, ** *p* < 0.01 and *** *p* < 0.001.

### 2.7. RNA Preparation and Quantitative Real-Time Polymerase Chain Reaction (qPCR)

Total RNA was extracted from sciatic nerves one day after nerve axotomy or SW10 cells with TRIzol reagent (Molecular Research Center, Cincinnati, OH, USA). We assessed the RNA quality with Agilent 2100 bioanalyzer (Agilent Technologies, Amstelveen, The Netherlands), and RNA quantification was performed using ND-2000 Spectrophotometer (Thermo-Fisher, Waltam, MA, USA). The concentration and purity of the isolated RNA samples were performed using a Nano Drop 2000 Spectrophotometer system; cDNA synthesis was carried out using QuantiMir kit (System Biosciences, Embarcadero, CA, USA) and Superscript III reverse transcriptase (Invitrogen, Waltham, MA, USA). The cycling conditions used for transcript detection were as follows: initial heating to 94 °C for 5 min, followed by cycling between 95 °C for 30 s 55 °C for 30 s, and 72 °C for 60 s for 40 cycles. For qPCR, the reaction was performed using TP700 (Takara, Otsu, Shiga, Japan). Primer sequences used in the validation were listed in Table S1.

### 2.8. Whole-Genome Profiling

RNA-seq libraries were prepared from total RNA using the NEBNext Ultra II Directional RNA-Seq Kit (New England BioLabs, Ipswich, MA, USA). The isolation of mRNAs was performed using the Poly(A) RNA Selection Kit (Lexogen, Wien, Austria). The isolated mRNAs were used for the cDNA synthesis and shearing, following the manufacturer’s instructions. Indexing was carried out using the Illumina indexes 1–12. The enrichment step was performed using PCR. Subsequently, the yield of cDNA libraries was quantified using the TapeStation HS D1000 Screen Tape (Agilent Technologies, Amstelveen, The Netherlands) to evaluate the mean fragment size. The size distribution of the cDNA libraries was determined using the library quantification kit using a StepOne Real-Time PCR System (Life Technologies, Carlsbad, CA, USA). High-throughput sequencing was performed as paired-end 100 sequencing using NovaSeq 6000 (Illumina, Sandiego, CA, USA).

### 2.9. miRNA Expression Profiling

For control and test RNAs, the construction of the library was performed using NEBNext Multiplex Small RNA Library Prep kit (New England BioLabs, Ipswich, MA, USA) according to the manufacturer’s instructions. Briefly, for library construction, total RNA from each sample was used at 1 μg to ligate the adaptors, and cDNA was synthesized using reverse-transcriptase with adaptor-specific primers. PCR was carried out library amplification, and libraries were performed clean-up using QIAquick PCR Purification kit (Quaigen, Hilden, Germany) and AMPure XP beads (Beckmancoulter, Brea, CA, USA). The yield and size distribution of the small RNA libraries were quantified using the Agilent 2100 Bioanalyzer instrument for the High-Sensitivity DNA Assay (Agilent Technologies, Santa Clara, CA, USA). High-throughput sequencing was produced by the NextSeq500 system as a way of single-end 75 sequencings (Illumina, Sandiego, CA, USA).

### 2.10. Bioinformatics

Mouse transcription factor and oxidative stress gene data were downloaded from the Animal Transcription Factor Database (https://bioinfo.life.just.edu.cm/AnimalTFDB/; accessed on 8 June 2022) and QuickGO (https://ebi.ac.uk/QuickGO/annotations; accessed on 8 June 2022). The DAVID bioinformatics functional annotation tool (https://david.ncifcrf.gov/home.jsp; accessed on 8 June 2022) was used to analyze gene ontology (GO) biological processes, Kyoto Encyclopedia of Genes and Genomes (KEGG) pathways, and Protein Family (PFAM) using differentially expressed genes, miRNAs, or proteins. Interaction networking among mRNAs, miRNAs, transcription factors, or oxidative stress genes/proteins was explored using the Cytoscape 3.7.0 (U.S National Institute of General Medical Sciences (NIGMS), Bethesda, MD, USA). We assigned them as nodes and links for statistical associations among the nodes. We also validated the interaction networking based on STRING (https://String-db.org; accessed on 8 June 2022) to provide information on the interaction among molecules.

### 2.11. Statistical Analysis

Statistical analysis was performed using Student’s *t*-test (non-paired two-tailed) to determine statistical significance. GraphPad Prism software (version 9.3.1, San Diego, CA, USA) was employed for statistical analysis. Differences were considered statistically significant for * *p* < 0.05, ** *p* < 0.01, *** *p* < 0.001.

## 3. Results

### 3.1. Whole-Genome Expression Profiling of Dedifferentiated Schwann Cells

We performed whole-genome expression profiling of DMSO-treated SW10 cells (control), as dedifferentiated Schwann cells and NEM-treated SW10 cells (H_2_S-pathway-inhibited Schwann cells), by RNA sequencing (RNA-seq) (Figure 1A). The duration of NEM treatment was selected in a time-dependent manner. After NEM treatment for 0, 3, 5, 6, 9, and 12 h, we counted the live cells at each time-point, and we selected 5 h for sampling the RNA-seq because the cell proliferation was significantly inhibited at 5 h in 10 µM NEM treated SW10 cells (Figure S1). We identified 293 upregulated and 447 downregulated differentially expressed genes (DEGs) between control and NEM-treated SW10 cells (Figure 1B). The fold changes of these DEGs are illustrated by a scatter plot (Figure 1C).

Among the upregulated genes, the top five enriched GO terms were response to oxidative stress, response to endoplasmic reticulum stress, smooth muscle cell proliferation, fibroblast growth factor stimulus, and apoptotic process (Figure 1D). KEGG pathways included MAPK, biosynthesis of cofactors, ferroptosis, p53, and cholesterol metabolism (Figure 1E). Enriched protein domains included bZIP transcription factor, Hsp70, amino acid permease, interferon-related protein conserved region, and degradation of Arg-rich protein for misfolding (Figure 1F). Among the downregulated genes, the top five enriched GO terms were cell cycle, transcription from RNA polymerase II promoter, multicellular organism development, cell proliferation, and chondrocyte differentiation (Figure 1G). Enriched KEGG pathways included cancer, rheumatoid arthritis, cytokine-receptor interaction, hepatitis B, and Wnt (Figure 1H). Enriched protein domains included yippee zinc-binding/DNA-binding/Mis18, TGF-β propeptide, transforming growth factor β-like domain, RFX1 transcription activation region, and kinesin motor protein (Figure 1I). The top 30 upregulated and downregulated genes were related to oxidative stress, cell cycle, and cholesterol metabolism (Table S2). The expression levels of 19 DEGs were validated at the mRNA level in SW10 cells by qPCR analysis (Figure 1J,K).

### 3.2. Transcriptional Regulation of Oxidative Stress in the H_2_S Signaling Pathway

Next, we determined the expression of a number of transcription factor genes expressed in sciatic nerves after nerve injury in vivo [12]. Of 48 transcription factor genes [13], 10—*Fosl1*, *Myc*, *Atf3*, *Cebpb*, *Fos*, *Mxd3*, *Cenpa*, *Runx2*, *Cenpt*, and *Zeb1*—significantly overlapped with the DEGs in the RNA-seq data (Figure 2A). Of those 10 genes, *Atf3* and *Fos* are expressed in Schwann cells during PND [14,15]. We validated the expression of eight genes in SW10 cells by qPCR (Figure 2B). To determine whether genes associated with oxidative stress, which ranked first among GO terms (Figure 1E), were associated with the H_2_S signaling pathway in Schwann cells, we compared their expression patterns between our RNA-seq data and those from the QuickGO database [16]. Of the 328 oxidative stress genes, 31 significantly overlapped with DEGs in our RNA-seq data (Figure 2C). Among the 31 oxidative stress genes, some are expressed in dedifferentiated Schwann cells, such as *Hmox1*, *Srxn1*, *Edn1*, and *Fos* [14,17,18,19]. Of those genes, nine were validated by qPCR using SW10 cells (Figure 1D).

**Figure 3:**
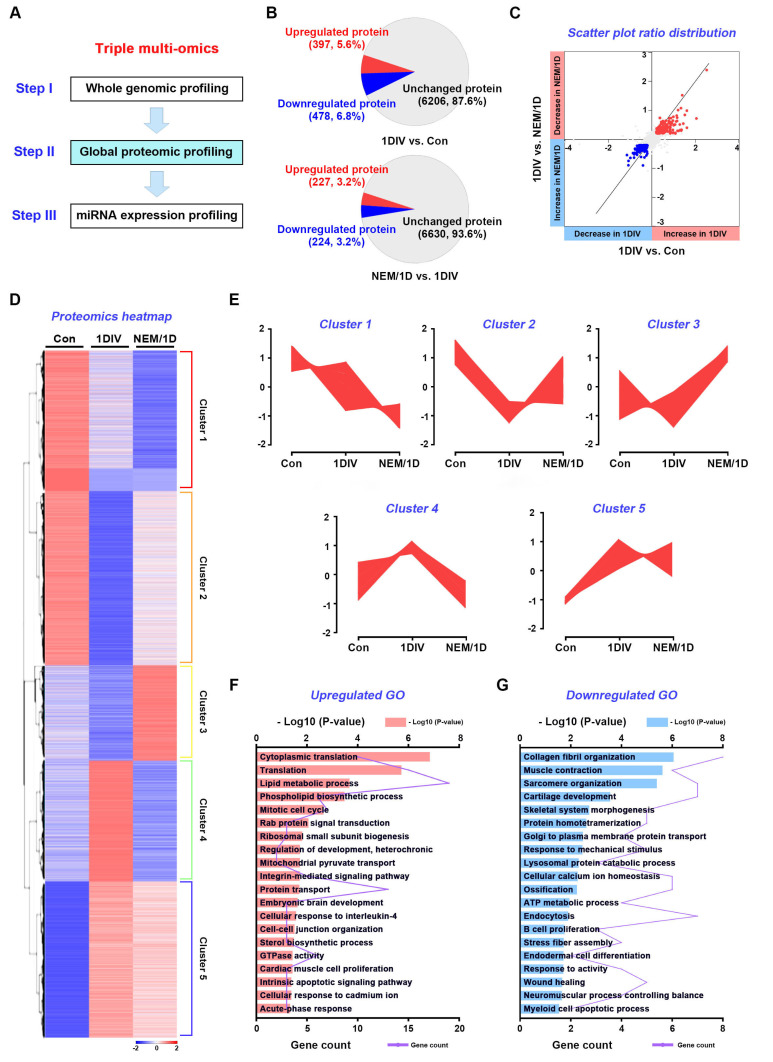
Global proteomics analysis with NEM as an inhibitor for hydrogen sulfide pathway. (**A**) The second stage of a three-stage framework for integrating triple multi-omics data. (**B**) The pie chart displays the numbers of upregulated (log_2_ FC > 0.2), downregulated (log_2_ FC < −0.2), and unchanged proteins in the total number of proteins (7149) among control mice nerves (non-injured) and ex vivo mice nerves with or without NEM for 1 day in vitro (1DIV). Control, con; NEM, *N*-ethylmaleimide; NEM/1D, NEM treatment for 1DIV. (**C**) The fold change (log2) among 7149 proteins in the nerves at 1DIV compared with control (1DIV/Con) is shown on the *x*-axis. The *y*-axis shows the change of each protein in the nerves with or without NEM at 1DIV comparison. The red, blue, and gray dots represent decreased proteins at NEM/1D, increased proteins at NEM/1D, and unregulated proteins, respectively. (**D**) Hierarchical clustering (1–5) of ex vivo sciatic nerves at 1DIV (1DIV) vs. control and NEM/1D vs. 1DIV. (**E**) Protein expression dynamic clusters based on global protein expression patterns among control, 1DIV, and NEM/1D. Gene ontology (GO) biological processes upregulated (**F**) and downregulated (**G**) in response to NEM treatment are shown.

Transcription factors regulate the expression of oxidative-stress-related genes [20,21]. In dedifferentiated Schwann cells, pathways between the 10 transcription factors and 31 oxidative stress genes were found unexpectedly. In the H_2_S signaling pathway, *Atf3* interacted with *Hmox1*, *Atf4*, *Gclm*, *Gclc*, *Plk3*, *Sesn2*, *Slc7a11*, *Srxn1*, *Stc2*, and *Txnip*. *Fos* interacted with *Hmox1*, *Atf4*, *Edn1*, *Apoe*, *Areg*, *Etv5*, *Pdgfra*, *Ptk2b*, *Snca*, *Star*, and *Bmp4* (Figure 1E). The oxidative stress and transcription factor DEGs are listed in Table S3.

### 3.3. Quantitative Proteomic Analysis of the H_2_S Signaling Pathway in Sciatic Nerves Ex Vivo

We performed a global quantitative proteomic profiling of ex vivo sciatic nerves, with or without NEM treatment, for 1 day in vitro (1DIV) (Figure 3A). We identified 7149 proteins in ex vivo sciatic nerves. There were 794 DE proteins (DEPs) (537 upregulated and 257 downregulated proteins) in ex vivo sciatic nerves at 1DIV (vs. control or 1DIV/control) and 305 (114 upregulated and 191 downregulated proteins) in ex vivo sciatic nerves at 1DIV with NEM treatment (NEM-treated 1DIV [NEM/1D] vs. 1DIV or NEM/1DIV); for DEPs, the ratio changed > 0.2-fold or < −0.2-fold (log2) (Figure 3B). The fold-changes of these DEPs were visualized as scatter plots (Figure 3C). Clustering heatmaps of quantitative proteins were constructed to assess protein expression patterns during ex vivo sciatic nerve degeneration, and the inhibitory effect of NEM on PND (Figure 3D). Five clusters (Clusters 1–5) were distinguished, comprising 1250, 2076, 1003, 1275, and 1652 proteins, respectively (Figure 3E). In Cluster 1, proteins expressed under normal conditions were decreased at 1DIV and further reduced upon NEM treatment. In Cluster 2, normally expressed proteins were drastically decreased at 1DIV and relatively recovered upon NEM treatment. In Cluster 3, proteins expressed at a low level under normal conditions or at 1DIV showed elevated expression in NEM-treated samples. In Cluster 4, proteins were highly expressed at 1DIV, but suppressed by NEM treatment. In Cluster 5, protein expression was similar between non- and NEM-treated conditions. GO terms in each cluster are shown in Figure S2 and DEPs are listed in Table S4 and Supplementary Material-2.

For NEM/1DIV, GO enrichment analysis showed that the upregulated DEPs were mainly significantly enriched in lipid metabolic process, phospholipid biosynthetic process, integrin-mediated pathway, cell–cell junction organization, and sterol biosynthetic process (Figure 3F). The downregulated DEPs were enriched in lysosomal protein catabolic process, endocytosis, B-cell proliferation, and endodermal cell differentiation (Figure 3G). In a KEGG pathway analysis, lipid and atherosclerosis, reactive oxygen species (ROS), lysosome, and diabetic cardiomyopathy were enriched (Figure S3A,B). The protein domains detected included the LIM domain, Ras family, fibrillar collagen C-terminal domain, and myosin tail (Figure S3C,D). Therefore, DEPs, via the H_2_S signaling pathway, modulate oxidative stress, myelination, and the cell cycle in ex vivo sciatic nerves during PND.

### 3.4. Interactions of Transcription Factors and Oxidative Stress Proteins in Sciatic Nerves Ex Vivo

**Figure 4:**
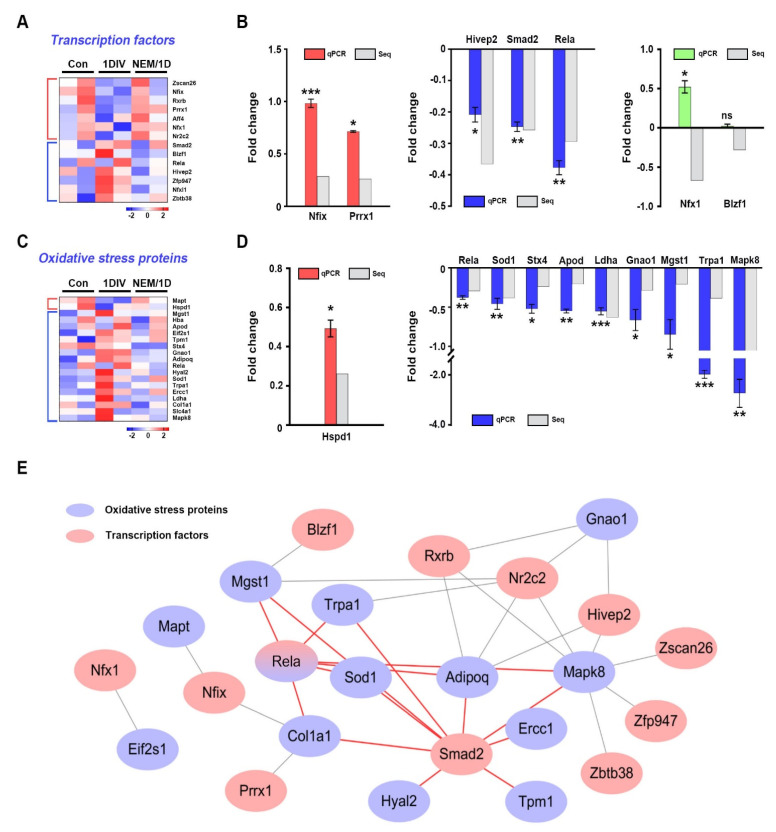
Interaction between oxidative stress proteins and transcription factors at the proteomic level. (**A**) Heatmap shows the upregulated and downregulated transcription factors in nerves between 1DIV and NEM/1D. (**B**) qPCR validation of the transcription factors obtained from (**A**) using ex vivo mouse sciatic nerves. Red bars, blue bars, and green bars represent upregulated, downregulated, and mixed genes compared with proteomics data. RNA-sequencing, Seq. (**C**) Heatmap was constructed for the significantly different oxidative stress proteins in nerves between 1DIV and NEM/1D. (**D**) Validation of significantly differentially expressed oxidative stress proteins using qPCR. One upregulated and eight downregulated oxidative stress proteins were validated. RNA-sequencing, Seq. (**E**) Interaction network between oxidative stress proteins and transcription factors. Among the connecting lines, the red lines indicate the interaction between oxidative stress proteins and transcription factors. Gray nodes represent proteins interacting with oxidative stress proteins. Comparisons were performed using a Student’s *t*-test. * *p* < 0.05, ** *p* < 0.01, and *** *p* < 0.001.

To identify transcription factors related to the H_2_S signaling pathway during ex vivo PND, we selected 14 DEPs among the NEM/1DIV data, because transcriptional regulation is essential for switching between myelinated and dedifferentiated Schwann cells (Figure 4A). Rela is one of five subtypes of NF-κB, a transcription factor associated with Schwann cell proliferation and myelination [22,23]. Smad2 regulates Schwann cell migration and proliferation [24]. In this study, seven proteins were validated at the mRNA level by qPCR analysis using control and ex vivo sciatic nerves, with or without NEM treatment at 6 h in vitro (6HIV) (Figure 4B). The Nifx1 and blzf1 expression levels may be dependent on post-transcriptional or translational regulation.

Next, because KEGG enrichment analysis identified ROS (Figure S3A), to test whether proteins involved in oxidative stress were regulated by NEM during ex vivo PND, we selected 19 oxidative stress proteins from the NEM/1DIV data overlapped with the QuickGO database [16] (Figure 4C). Among them, Sod1, Rela, Gnao1, Stx4, Eif2s1, and Mapt regulate Schwann cell proliferation, migration, and differentiation [23,24,25,26,27,28]. The expression of 10 proteins was validated at the mRNA level by qPCR using ex vivo sciatic nerves (Figure 4D). Rela and Smad2 interacted with six and nine oxidative stress proteins, respectively (Figure 4E). Rela interacted with AdipoQ, Col1a1, Mapk8, Mgst1, Sod1, and Trpa1. Smad2 interacted with AdipoQ, Col1a1, Ercc1, Hyal2, Mapk8, Mgst1, Sod1, Tpm1, and Trpa1. Therefore, Rela and Smad2 may be involved in the NEM-dependent transcriptional regulation of oxidative stress proteins in the H_2_S signaling pathway during PND.

### 3.5. miRNA Expression Profiling of the H_2_S Signaling Pathway in Dedifferentiated Schwann Cells

**Figure 5:**
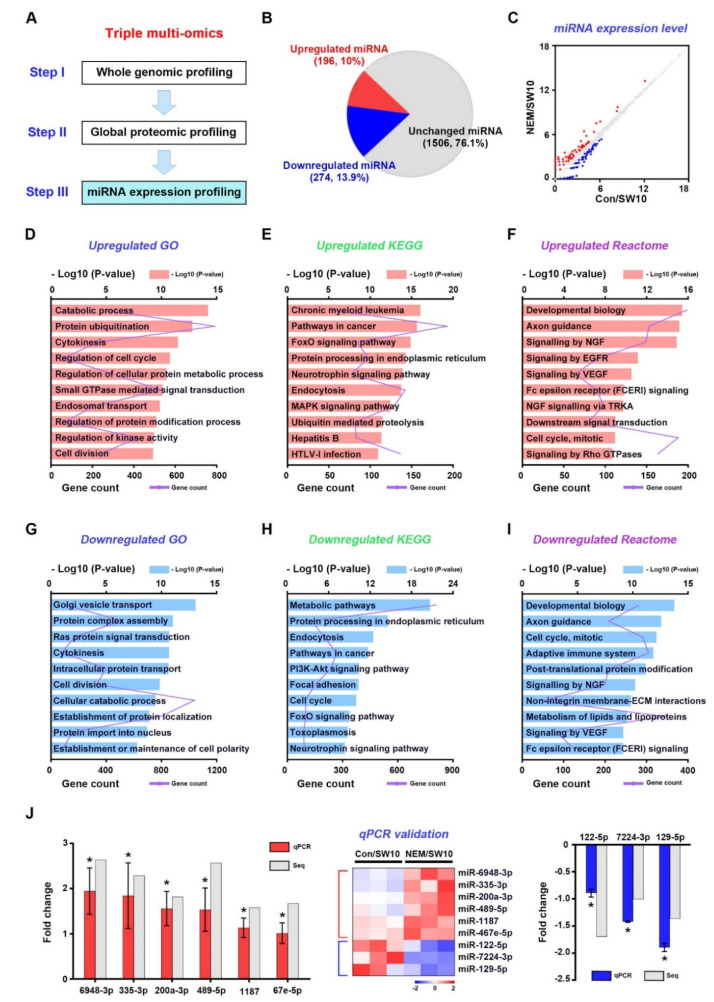
miRNA expression profiling with NEM as an inhibitor for hydrogen sulfide pathway. (**A**) The third stage of a three-stage framework for integrating triple multi-omics data. (**B**) A pie chart displays 10% upregulated and 13.9% downregulated miRNAs in the total number of miRNAs (1976) between DMSO- and NEM-treated SW10 cells. (**C**) A scatter plot of the comparison of miRNA expression between DMSO- and NEM-treated SW10 cells. Upregulated and downregulated miRNAs are indicated by red dots in the upper region and blue dots in the lower region, respectively. Using upregulated miRNAs, GO terms (**D**), KEGG pathways (**E**), and Reactome analysis (**F**) are shown. Using downregulated miRNAs, GO terms (**G**), KEGG pathways (**H**), and Reactome analysis (**I**) are shown. (**J**) qPCR validation of top 5 upregulated and downregulated miRNAs. The log2 FC was shown in both RNA-sequencing (Seq) and the validation. Heatmap shows the relative expression levels of miRNAs. Comparisons were performed using a Student’s *t*-test. * *p* < 0.05.

To identify post-transcriptional regulation via the H_2_S signaling pathway in dedifferentiated Schwann cells, we performed a small RNA-seq (Figure 5A). High-throughput expression analysis of 800 miRNAs was performed for control and NEM-treated SW10 cells. The analysis yielded 470 differentially expressed (DE) miRNAs; among them, 196 DE were upregulated, and 274 DE were downregulated, in SW10 cells (Figure 5B,C).

Next, we performed GO term, KEGG pathway, and reactome pathway analyses of DE miRNA functions. Among the upregulated DE miRNAs, the top five GO enrichment terms were catabolic process, protein ubiquitination, cytokinesis, cell cycle, and cellular protein metabolic process (Figure 5D); the top five KEGG pathways were chronic myeloid leukemia, pathways in cancer, FoxO pathway, protein process in endoplasmic reticulum, and neurotrophin pathway (Figure 5E); and the top five reactome pathways were developmental biology, axon guidance, signaling by NGF, signaling by EGFR, and signaling by VEGF (Figure 5F). Among the downregulated DE miRNAs, the top five GO enrichment terms were Golgi vesicle transport, protein complex assembly, Ras protein signal transduction, cytokinesis, and intracellular protein transport (Figure 5G); the top five KEGG pathways were metabolic pathways, protein process in endoplasmic reticulum, endocytosis, pathways in cancer, and PI3K–Akt pathway (Figure 5H); and the top five reactome pathways were developmental biology, axon guidance, cell cycle, adaptive immune system and post-translational protein modification (Figure 5I). Therefore, the target genes of DE miRNAs are related to catabolic process, the cell cycle, and neurotrophin signaling. The expression levels of nine miRNAs in SW10 cells were validated by qPCR (Figure 5J). The top 30 upregulated and downregulated miRNAs are listed in Table S5.

### 3.6. Post-Transcriptional Regulation of the Response to Oxidative Stress

**Figure 6:**
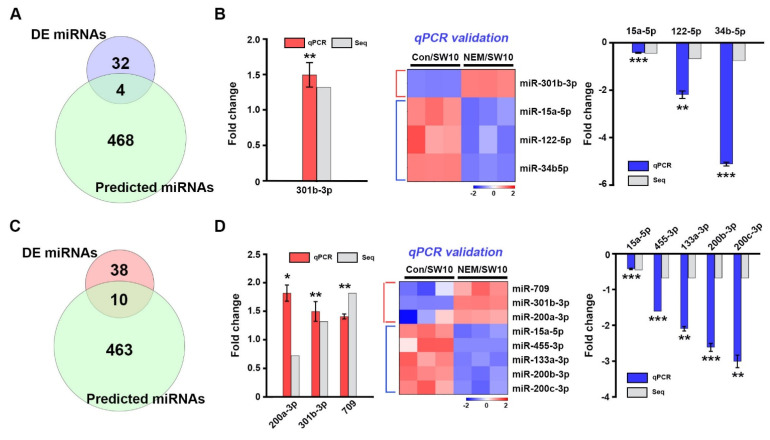
Interaction among oxidative stress genes, transcription factors, and miRNAs in the post-transcriptional regulation (**A**) Venn diagram analysis of differentially expressed (DE) miRNAs from our RNA-sequencing (Seq) and miRNAs targeting oxidative stress genes predicted by miRWalk2.0. (**B**) One upregulated miRNA and three downregulated miRNAs were validated by qPCR. Heatmap shows the relative expression levels of the miRNAs. (**C**) Venn diagram analysis of differentially expressed (DE) miRNAs from our RNA-sequencing (Seq) and miRNAs targeting transcription factor genes predicted by miRWalk2.0. (**D**) Three upregulated miRNAs and five downregulated miRNAs were validated by qPCR. Heatmap shows the relative expression levels of the miRNAs. Comparisons were performed using a Student’s *t*-test. * *p* < 0.05, ** *p* < 0.01 and *** *p* < 0.001.

To assess the post-transcriptional regulation of oxidative stress and catabolic process genes by miRNAs in SW10 cells, we screened miRNAs targeting 50 genes detected by proteomics and mRNA-seq (Figure 6A). Prediction of miRNA targeting was performed by miRWalk2.0 [29]; miR-301b-3p targeted *Etv5* and *Slc7a11*; *Txnip* was a target of both miR-301b-3p and miR-15a-5p; miR-15a-5p, miR-24b-5p, and miR122-5p targeted *Aldoa*. The expression of four miRNAs was validated by qPCR (Figure 6B). We performed an interaction analysis between the four miRNAs and their targets (Figure S4).

Next, we evaluated the post-transcriptional regulation of transcription factors associated with oxidative stress and catabolic process genes in SW10 cells. We selected 24 transcription factor genes from the proteomics and mRNA-seq data (Figure 6C). We compared our miRNA-seq data with the predicted miRNAs of 24 transcription factor genes using miRWalk2.0; 10 miRNAs were identified as post-transcriptional regulators. Of these, miR-200a-3p, miR-200b-3p, and miR-200c-3p targeted *Zeb1*, and miR-401a and miR-709 targeted *Myc*; *Smad2* was targeted by miR-15a-5p and miR-455-3p; miR-301b-3p and miR-133a-3p targeted *Nfix* and *Runx2*, respectively; *Zfp947*, *Nfx1*, and *Nr2c2* were targeted by miR-15a-5p, and *Aff4* was targeted by miR-297a-5p and miR-301b-3p. Nine miRNAs were validated by qPCR in SW10 cells (Figure 6D). The interconnection network between the 10 miRNAs and 24 transcription factors is highlighted in Figure S5. Therefore, oxidative stress/catabolic process genes and transcription factors related to oxidative stress factors in the H_2_S signaling pathway are post-transcriptionally regulated by miRNAs in Schwann cells during PND.

## 4. Discussion

The H_2_S signaling pathway regulates a variety of physiological and pathological conditions in mammalian cells [30,31,32]. H_2_S, as an endogenous modulator, readily diffuses and reacts with numerous molecules in living cells. Mammalian cells generate H_2_S via enzymatic and non-enzymatic pathways, such as by reducing persulfides, thiosulfides, and polysulfides. CBS and CSE (pyridoxal 5′-phosphate [PLP]-dependent enzymes) are located in the cytosol, and MST (a PLP-independent enzyme) is located in mitochondria. Interestingly, under oxidative stress, CBS and CSE translocate into mitochondria and compensate for MST inactivation [33]. Thus, the H_2_S signaling pathway is an important component of the response to oxidative stress. H_2_S protects cells against oxidative stress via two mechanisms. First, because oxidative stress is associated with molecular and cellular damage caused by ROS and reactive nitrogen species (RNS), deficiency of antioxidants is a plausible mechanism. In this scenario, H_2_S acts as a direct scavenger of ROS or RNS. Alternatively, antioxidant enzyme systems are disrupted, thus altering the cellular reduction–oxidation balance and increasing the expression of antioxidant enzymes. In the PNS, oxidative stress is a major cause of PND [8], and its deleterious effects can be prevented by depleting antioxidants and antioxidant enzymes.

Methodology to study Schwann cells has been implemented with various methods. Among them, omics analysis allows a sophisticated study of Schwann cells. Shen et al. showed a proteome map in primary Schwann cell culture [34], and Weiss analyzed the proteome of peripheral nerve tissue [35]. Arthur-Farraj et al. performed the coding and non-coding transcriptome and DNA methylome using peripheral nerves [36], and Schira et al. analyzed secretomes derived from primary Schwann cell cultures [37]. However, H2S-pathway-associated multi-omics approaches were performed for the first time in this study. In the NEM-treated Schwann cells, suppression of the H2S signaling pathway was seen [8]. In this condition, transcriptome and proteome functional enrichment analyses consistently showed involvement in oxidative stress, lipid metabolic process (myelination), and the cell cycle. In the mRNA-seq and miRNA-seq analyses, the response to oxidative stress and catabolic process GO terms were enriched, respectively (Figure 1D and Figure 5D). The KEGG pathways enriched in the mRNA-seq and proteomics analyses were ferroptosis and ROS, respectively (Figure 1E and Figure S3A). Interestingly, at the transcriptional and post-transcriptional levels, the above terms were listed first (Figure 3D and Figure 5D). Catabolic processes break down molecules, for example via oxidation [38]. Thus, NEM-induced responses to oxidative stress may affect—in a manner dependent on the H2S pathway—the demyelination and dedifferentiation of Schwann cells during PND. Schwann cell myelination involves lipid metabolism, and oxidative stress reduces Schwann cell plasticity and demyelination [18,39,40]. The KEGG pathways showing enrichment in the mRNA-seq and proteomics analyses included cholesterol metabolism and lipid and atherosclerosis (Figure 1E and Figure S3A). In the miRNA-seq analysis, metabolism of lipids and lipid proteins were highly ranked reactome pathways (Figure 5I). In the proteomics analysis, lipid metabolic process, phospholipid biosynthetic process, and sterol biosynthetic process were enriched GO terms (Figure 3F). Schwann cell proliferation is affected by oxidative stress in a manner dependent on the H_2_S signaling pathway. Oxidative stress inhibits Schwann cell differentiation and proliferation [17,41,42]. In this study, many genes showed functional enrichment involving cell proliferation (Figure 1, Figure 3, and Figure 5). In the mRNA-seq data, enriched GO terms and KEGG pathways included cell cycle, cell proliferation, cell division, and cell-cycle-related signaling pathways (Figure 1D,E,G,H). Moreover, miRNA-seq showed GO, KEGG, and reactome enrichment of cell cycle, cell proliferation, and cell division (Figure 5D–I). According to proteomics analysis, mitotic cell cycle and cardiac/B cell/endodermal/muscle cell proliferation were enriched GO terms (Figure 3F,G). Therefore, the response of the H_2_S pathway to oxidative stress could affect myelination via lipid metabolism, and proliferation via cell cycle/cell division, in Schwann cells during PND.

The deleterious effects of oxidative stress can be prevented by the upregulation of antioxidant enzymes and their transcription factors. In molecular network analyses (Figures S4 and S5), all 24 transcription factors from the proteomics and mRNA-seq data involved in the response to oxidative stress were targeted by 10 mmu-miRNAs (Figure 6C and Figure S5). Among them, four miRNAs (miR-451a, miR-301b-3p, miR-709, and miR-200a-3p) were upregulated and six (miR-297a-5p, miR-15a-5p, miR-133a-3p, miR-200b-3p, miR-200c-3p, and miR-455-3p) were downregulated. Interestingly, Smad2 was the most important transcription factor at the proteomics level (Figure 4C); it interacted with several oxidative stress proteins, suggesting a role in maintaining the oxidation–reduction balance. *Smad2* may be controlled by post-transcriptional regulation via miR-15a-5p and miR455-3p during PND (Figure S5). Therefore, suppression of PND by NEM influenced transcription factors, such as *Smad2*, regulating the expression of oxidative stress-related genes in Schwann cells.

Schwann cell dedifferentiation is associated with lysosomal activity, cell migration, and neurotrophin [43,44]. In this study, neurotrophin and NGF signaling were highlighted by the miRNA-seq analysis (Figure 3E,F,H,I). Lysosomes, cell–cell junction organization, and lysosomal protein catabolic process were highlighted by the proteomics analysis (Figure 3F,G and Figure S3B). The MAPK, PI3K–Akt, and Wnt pathways are associated with Schwann cell dedifferentiation [45,46] and were highlighted by mRNA-seq functional enrichment analysis (Figure 1E,H). Therefore, NEM-induced Schwann cell dedifferentiation may be regulated by the H_2_S-pathway, via various biochemical events occurring at the transcriptional, post-transcriptional, and translational levels during PND.

## 5. Conclusions

We performed a triple multi-omics analysis of *in vitro*- and *ex vivo*-dedifferentiated Schwann cells to assess the pharmacological effect of NEM and the molecular mechanism of the H_2_S pathway in PND. In a previous study, NEM-induced H_2_S pathway inhibition in Schwann cells suppressed PND. Here, the NEM-mediated regulation of oxidative stress was dependent on transcription factors in the H_2_S signaling pathway, such as Rela, Fos, Smad2, and Atf3. The multi-omics analysis suggested molecular or pharmacological targets for regulating the neurodegeneration of Schwann cells during PND. Our findings will facilitate the development of new therapeutics for intractable peripheral neurodegenerative diseases.

## Data Availability

Not applicable.

## Supplementary Materials

The following supporting information can be downloaded at: https://www.mdpi.com/article/10.3390/antiox11081606/s1, Figure S1: Inhibition patterns of SW10 cell proliferation in a time-dependent manner; Figure S2: Functional gene ontology (GO) at protein expression dynamic cluster 1–5 in global proteomics analysis among control, 1DIV, and NEM/1D; Figure S3: Kyoto Encyclopedia of Genes and Genomes (KEGG) and Protein Family (PFAM) using differentially expressed proteins (DEPs) between NEM/1D and 1DIV; Figure S4: Complexity between the miRNAs and oxidative stress gene network; Figure S5; Interaction between miRNAs and transcription factor gene network; Table S1: List of primer sequences used for qPCR; Table S2: List of top 30 upregulated and downregulated genes; Table S3: List of oxidative stress and transcription factor genes; Table S4: List of top 30 upregulated and downregulated proteins; Table S5: List of top 30 upregulated and downregulated miRNAs.
