# Supplementary material for: Investigation of the Hydrogen Sulfide Signaling Pathway in Schwann Cells during Peripheral Nerve Degeneration: Multi-Omics Approaches
Source: Antioxidants (Basel). 2022 Aug 19;11(8):1606. doi: 10.3390/antiox11081606 (PMC9405209; doi:10.3390/antiox11081606)
Supplement: Supplementary file 1 [file antioxidants-11-01606-s001.zip › antioxidants-1838690-Supplementary Material.pdf]

Supplementary Materials

Investigation of the Hydrogen Sulfide Signaling Pathway in Schwann Cells During Peripheral Nerve Degeneration: Multi-omics Approaches

Yoo Lim Chun[1,2,3†], Won-Joon Eom[1,2†], Jun Hyung Lee[4†], Thy N. C. Nguyen[4], Ki-Hoon Park[1,5], Hyung-Joo Chung[5], Han Seo[5], Youngbuhm Huh[1,2], Sang Hoon Kim[6], Seung Geun Yeo[6], Wonseok Park[7], Geul Bang[8], Jin Young Kim[8], Min-Sik Kim[4]*, Na Young Jeong[3]*, Junyang Jung[1,2]*

[†]These authors contributed equally to this work

[1]Department of Anatomy and Neurobiology, College of Medicine, Kyung Hee University, Dongdaemun-gu, Seoul 02447, Korea.
[2]Department of Biomedical Science, Graduation School, Kyung Hee University, Dongdaemun-gu, Seoul 02447, Korea.
[3]Department of Anatomy and Cell Biology, College of Medicine, Dong-A University, Seo-gu, Busan 49201, Korea.
[4]Department of New Biology, Daegu Gyeongbuk Institute of Science and Technology (DGIST), Dalseong-gun, Daegu 42988, Korea.
[5]Department of Anesthesiology and Pain Medicine, College of Medicine, Kosin University, Seo-gu, Busan 49267, Korea.
[6]Department of Otorhinolaryngology-Head and Neck Surgery, College of Medicine, Kyung Hee University, Dongdaemun-gu, Seoul 02447, Korea.
[7]Department of Orthopedic Surgery, Good Samsun Hospital, Sasang-gu, Busan, 47007, Korea.
[8]Research Center for Bioconvergence Analysis, Korea Basic Science Institute, Ochang 28119, Republic of Korea

***Correspondences should be addressed to:**

Min-Sik Kim Ph.D. E-mail: mkim@dgist.ac.kr
Na Young Jeong MD, Ph.D. E-mail: jnyjjy@dau.ac.kr
Junyang Jung MD, Ph.D. E-mail: jjung@khu.ac.kr

Contents

Supplementary Figure S1. Inhibition patterns of SW10 cell proliferation in a time-dependent manner.

Supplementary Figure S2. Functional gene ontology (GO) at protein expression dynamic clusters 1-5 in global proteomics analysis among control, 1DIV, and NEM/1D.

Supplementary Figure S3. Kyoto Encyclopedia of Genes and Genomes (KEGG) and Protein Family (PFAM) using differentially expressed proteins (DEPs) between NEM/1D and 1DIV.

Supplementary Figure S4. Complexity between the miRNAs and oxidative stress gene network.

Supplementary Figure S5. Interaction between the miRNAs and transcription factor gene network.

Supplementary Table S1. List of primer sequences used for qPCR.

Supplementary Table S2. List of top 30 upregulated and downregulated genes.

Supplementary Table S3. List of oxidative stress and transcription factor genes.

Supplementary Table S4. List of top 30 upregulated and downregulated proteins.

Supplementary Table S5. List of top 30 upregulated and downregulated miRNAs.

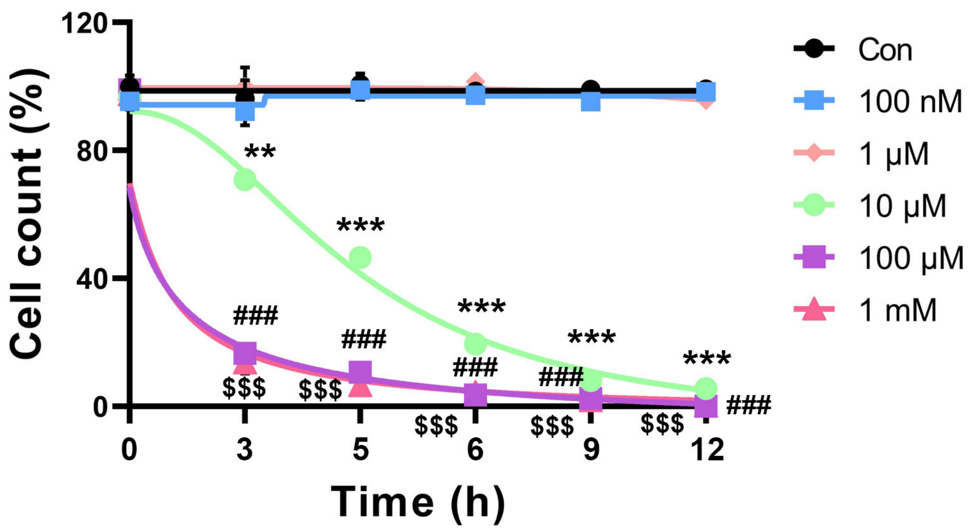

Figure S1. Inhibition patterns of SW10 cell proliferation in a time-dependent manner. SW10 cell were treated with *N*-ethylmaleimide (NEM) at several concentrations (100 nM, 1 μM, 10u M, 100 uM, 1 mM) and observed in each time-course. Live cells of each group were counted and shown as line graphs**.** **P < 0.01 and ***, ###, and $$$P < 0.001.

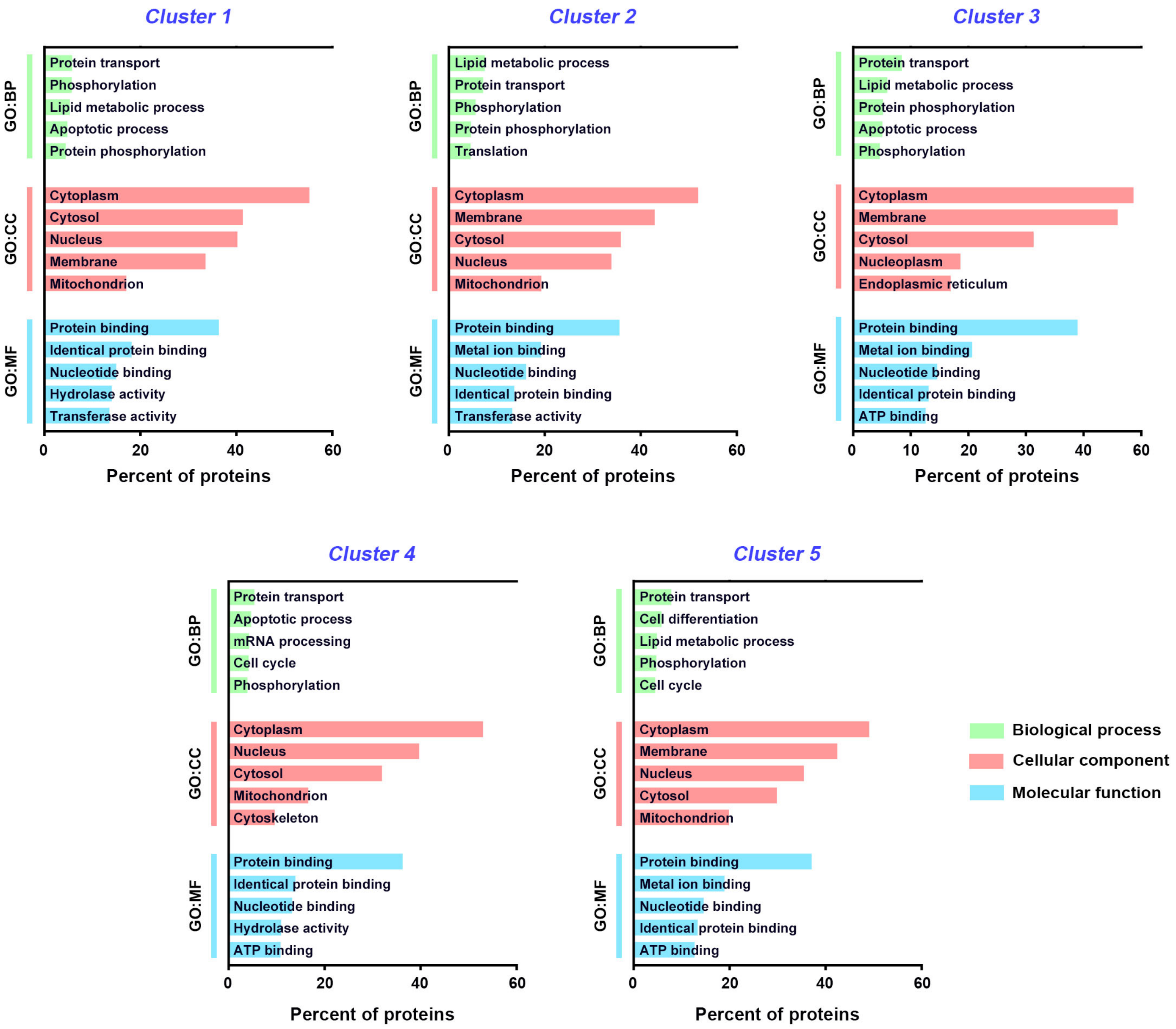

Figure S2. Functional gene ontology (GO) at protein expression dynamic clusters 1-5 in global proteomics analysis among control, 1DIV, and NEM/1D. The functional GO terms were shown at each cluster. BP, biological process; CC, cellular component; MF, molecular functions. 1DIV, nerves with no treatment at 1 day *in vitro*; NEM1/D, nerves with NEM treatment at 1DIV.

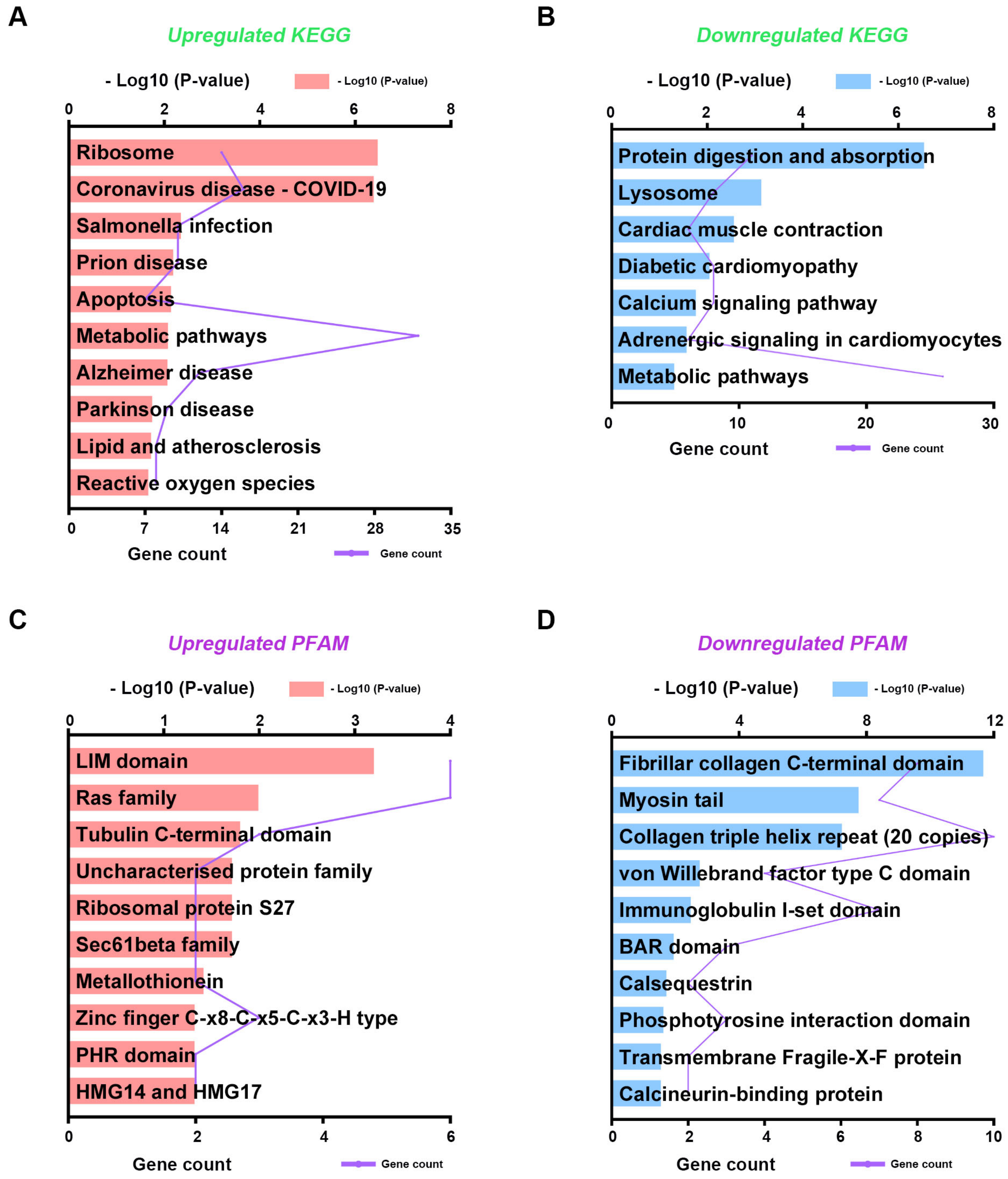

Figure S3. Kyoto Encyclopedia of Genes and Genomes (KEGG) and Protein Family (PFAM) using differentially expressed proteins (DEPs) between NEM/1D and 1DIV. KEGG pathways upregulated and downregulated in response to NEM treatment were shown in (**A**) and (**B**), **r**espectively. PFAM upregulated and downregulated in response to NEM treatment was shown in (**C**) and (**D**), respectively.

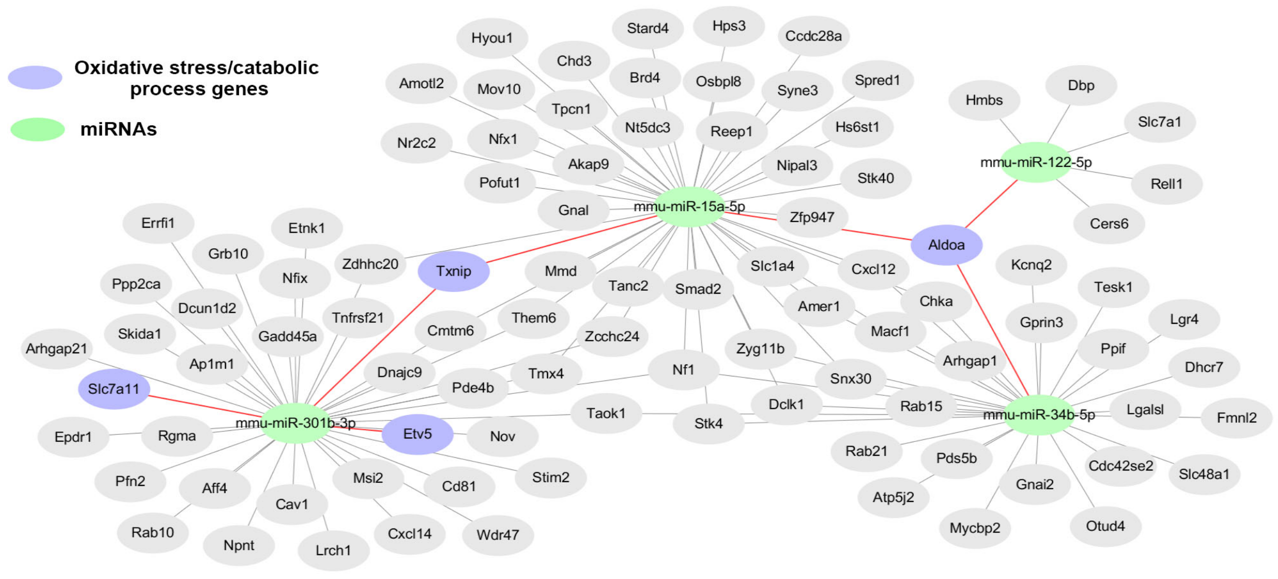

Figure S4. Complexity between the miRNAs and oxidative stress gene network. Green and purple dots represented miRNAs and oxidative stress genes. The red line represented commonly interconnected miRNAs and oxidative stress genes.

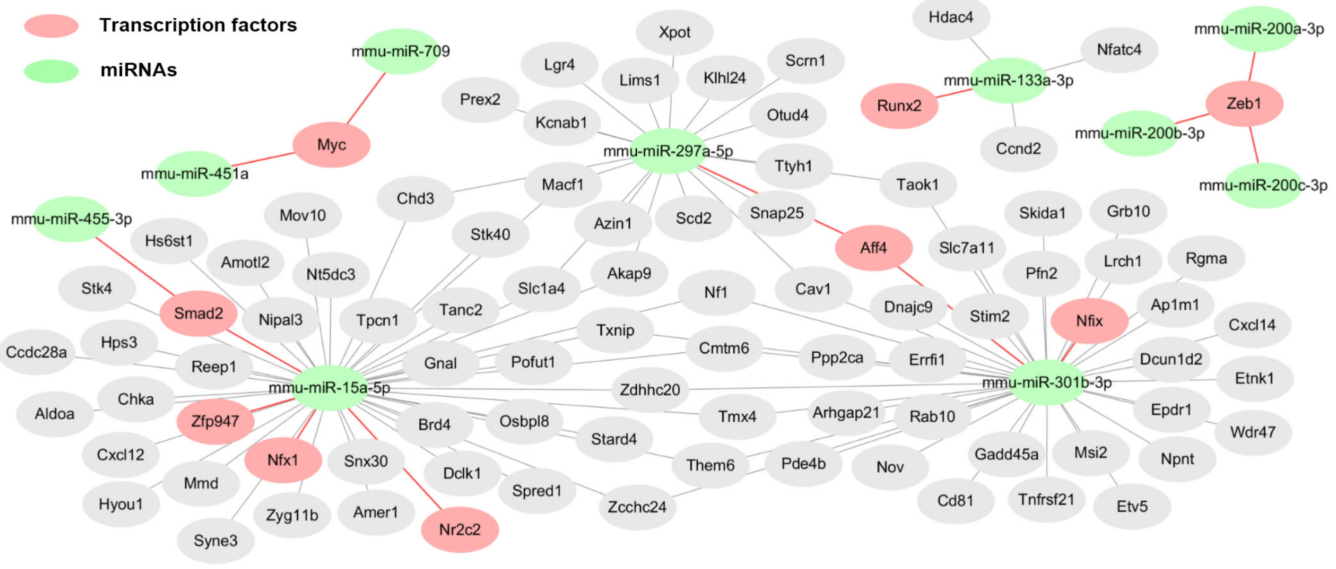

Figure S5. Interaction between the miRNAs and transcription factor gene network. Green and red dots represented miRNAs and transcription factor genes. Interaction networking was explored using the Cytoscape 3.7.0.

Table S1. List of primer sequences used for qPCR

Primer Name	Primer sequence (5' to 3')	Primer Name	Primer sequence (5' to 3')
Nfix	Forward - CTTCCACCAGCAGCACCAA	Fosl1	Forward - GCATGTACCGAGACTACGGGG
	Reverse - GGGCTCCCTGCATCCAC		Reverse - TGCACCATCCAGTGCAGTTCCT
Prrx1	Forward - AAGAACTTCTCCGTCAGTCACCT	Slc7a11	Forward - ATACTCCAGAACACGGGCAG
	Reverse - CCTGTTTCTCCGCTGCTTTCT		Reverse - AGTTCCACCCAGACTCGAAC
Rela	Forward - CGGGATGGCTACTATGAGG	Herpub1	Forward - AGAACATCTCTAGGCCTGAG
	Reverse - CTCCAGGTCTCGCTTCTT		Reverse - TGCCTTGCATAGATCTGCTG
Smad2	Forward - GTAAGATCCCACCAGGCTGTAA	Ddit3	Forward - ACAGAGCCAGAATAACAGCCG
	Reverse - CTCCCCAGCCCTTCACAAAA		Reverse - TCTGCTTTCAGGTGTGGTGGTG
Hivep2	Forward - CCCCACGTTTTACAGCAAGAGC	Srxn1	Forward - GACGTCCTCTGGATCAAAG
	Reverse - TCTGAAAGTCCACCCGCTTCTC		Reverse - GCAGGAATGGTCTCTCTCTG
Nfxl1	Forward - AGAACCTCCTCAGTTGCTGC	Col3a1	Forward - TGCTCCTGTGCTTCCTG
	Reverse - AAGGGGCATTCACCAGGATG		Reverse - GACCTGGTTGTCCTGGA
Blzf1	Forward - CAGGCAGAAGTGGTCACAGA	Tns1	Forward - CTGCCCCTTGCGTTCCT
	Reverse - AGTGATACTGCGGGTCATCC		Reverse - ACTTCCAACCCGGCAGTCT
Hspd1	Forward - CACAGTCCTTCGCCAGATGAG	Fos	Forward - TTCCACCCCAGAGTCTGAGGA
	Reverse - CTACACCTTGAAGCATTAAGGCT		Reverse - GCTCCACGTTGCTGATGCTC
Stx4	Forward - CCTGCAGAACCTGCGAGAGGAGAT	Wisp2	Forward - GTACCTGGATGGGGAGACCT
	Reverse - CCTCCGGATCCGCTCCACATT		Reverse - CACCTTCCTGGCACCTGTAT
Sod1	Forward - GCAGGGAACCATCCACTTCG	Tnbs1	Forward - TGATGACTACGCTGGCTTTG
	Reverse - CCTGCACTGGTACAGCCTTG		Reverse - TGAGTATCCCTGAGCCCTTG
Trpa1	Forward - CAGGATGCTACGGTTTTTTCATTACT	Rgcc	Forward - GGCTTCAGCGACTCGGAG
	Reverse - GCATGTGTCAATGTTTGGTACTTCT		Reverse - CTTTCCGAGGAGTGACAGCG
Ldha	Forward - GGCACTGACGCAGACAAG	Slc40a1	Forward - GCTGCTAGAATCGGTCTTTGGT
	Reverse - TGATCACCTCGTAGGCACTG		Reverse - CAGCAACTGTGTCACCGTCAA
Gnao1	Forward - TACTACCTGGACAGCCTGGA	Gapdh	Forward - CGTCCCGTAGACAAAATGGT
	Reverse - GGATCCACTTCTTGCGTTCA		Reverse - TTGATGGCAACAATCTCCAC
Mgst1	Forward - TTTTGCCAACCCAGAAGACT	Cenpd	Forward - CGCGTTCATGCACCGCCTGC
	Reverse - GAGGCCGATGCCGAGAAAGG		Reverse - CCAGGCAGTCGGGCTCGTAGTAG
Mapk8	Forward - GCCACAAAATCCTCTTTCCA	Atf3	Forward - AAGGGGTGATGCAACGCGCT
	Reverse - CACATCGGGGAACAGTTTCT		Reverse - CGCGGGTTAGCCGATTGGCT
Hmox1	Forward - CTCATCCCAGACACCGC	Mxd3	Forward - AAGATTTGGTGGTGCCTGAA
	Reverse - CAGGCAAGATTCTCCCTTACAGA		Reverse - CCTTTGTGGCTTTGTGGTTGT
Trib3	Forward - GGCCTTATATCCTTTTGGAACGA	Zeb1	Forward - ACCCCTTCAAGAACCGCTTT
	Reverse - CGCTGGCAGGGTACACCTT		Reverse - CAATTGGCCACCACTGCTAA
Stc2	Forward - AGGAGAACGTCGGTGTGATT	Cenpt	Forward - GAACATGGCGGACCTCAG
	Reverse - CTGTTCACACTGAGCCTGGA		Reverse - AGTGCTCCGGCGTCTCAT
Slco4a1	Forward - TTACTGCCTGTCCTGGAAGC	Txnip	Forward - AGTGATTGGCAGCAGGTC
	Reverse - TCAGTGCAGTTTGCTTGGAC		Reverse - GGTATCTGGGATGTTTAGG
Cxcl12	Forward - ATCCTCAACACTCCAAACTGTGCC	Myc	Forward - TTGGAAACCCCGCAGACAG
	Reverse - TTCAGACCTAGGCTCCTCCTGTAA		Reverse - GCTGTACGGAGTCGTAGTCG
Cxcl1	Forward - AGACCATGGCTGGGATTCAC	Bmp4	Forward - TGAGTACCCGGAGCGTCC
	Reverse - AGCTTCAGGGTCAAGGCAAG		Reverse - CTCCAGATGTTCTTCGTGATGG
Apod	Forward - GAAGCCAAACAGAGCAACG		
	Reverse - TGTTTCTGGAGGGAGATAAGGA		
mmu-miR-U6	TGGCCCCTGCGCAAGGATG	mmu-miR-129-5p	CTTTTTGCGGTCTGGGCTTGC
mmu-miR-6948-3p	TGTCCTTCCTGTCTGACCACA	mmu-miR-301b-3p	CAGTGCAATGGTATTGTCAAAGC
mmu-miR-335-3p	TTTTTCATTATTGCTCCTGACC	mmu-miR-15a-5p	TAGCAGCACATAATGGTTTGTG
mmu-miR-200a-3p	TAACACTGTCTGGTAACGATGT	mmu-miR-34b-5p	AGGCAGTGTAATTAGCTGATTGT
mmu-miR-489-5p	TGTCATATGTGTGATGACACTTTCT	mmu-miR-709	GGAGGCAGAGGCAGGAGGA
mmu-miR-1187	TATGTGTGTGTGTATGTGTGTAA	mmu-miR-455-3p	GCAGTCCACGGGCATATACAC
mmu-miR-467e-5p	ATAAGTGTGAGCATGTATATGT	mmu-miR-133a-3p	TTTGGTCCCCTTCAACCAGCTG
mmu-miR-122-5p	TGGAGTGTGACAATGGTGTTTG	mmu-miR-200b-3p	TAATACTGCCTGGTAATGATGA
mmu-miR-7224-3p	TCCACTGAGAGGACCACCCAC	mmu-miR-200c-3p	TAATACTGCCGGGTAATGATGGA

Table S2. List of top 30 upregulated and downregulated genes

Entrez ID	Gene Symbol	Log_2FC	up/down	Entrez ID	Gene Symbol	Log_2FC	up/down
15368	Hmox1	2.64	up	100037283	Rnaset2a	-2.42	down
228775	Trib3	2.57	up	69994	Rsc1a1	-2.06	down
26570	Slc7a11	2.55	up	14825	Cxcl1	-1.91	down
14283	Fosl1	2.30	up	319159	Hist1h4j	-1.90	down
504193	Npcd	2.29	up	53945	Slc40a1	-1.70	down
76650	Srxn1	2.29	up	66214	Rgcc	-1.70	down
11987	Slc7a1	2.22	up	319160	Hist1h4k	-1.70	down
12869	Cox8b	2.22	up	21961	Tns1	-1.70	down
20856	Stc2	2.22	up	14281	Fos	-1.70	down
64209	Herpud1	2.22	up	20315	Cxcl12	-1.56	down
100503710	Gm5741	2.01	up	97122	Hist2h4	-1.45	down
13198	Ddit3	1.87	up	21825	Thbs1	-1.45	down
108115	Slco4a1	1.87	up	22403	Wisp2	-1.45	down
394435	Ugt1a6b	1.87	up	171543	Bmf	-1.45	down
319520	Dusp4	1.87	up	319157	Hist1h4f	-1.45	down
69065	Chac1	1.87	up	12825	Col3a1	-1.45	down
381990	Zbtb2	1.87	up	75555	1700020D05Rik	-1.45	down
17869	Myc	1.87	up	241525	Ypel4	-1.45	down
22782	Slc30a1	1.87	up	319182	Hist1h2bh	-1.45	down
17768	Mthfd2	1.82	up	14282	Fosb	-1.45	down
18793	Plaur	1.82	up	208990	Npb	-1.45	down
71839	Osgin1	1.82	up	326620	Hist1h4b	-1.45	down
17313	Mgp	1.82	up	218203	Mylip	-1.45	down
68223	Fam24a	1.82	up	76933	Ifi27l2a	-1.45	down
20361	Sema7a	1.70	up	14102	Fas	-1.40	down
17386	Mmp13	1.67	up	13865	Nr2f1	-1.37	down
215814	Ccdc28a	1.67	up	12159	Bmp4	-1.37	down
11816	Apoe	1.62	up	73363	1700056E22Rik	-1.37	down
16975	Lrp8	1.58	up	23967	Osr1	-1.37	down
11717	Ampd3	1.42	up	21810	Tgfbi	-1.26	down

Table S3. List of oxidative stress and transcription factor genes

Associated Gene	Entrez ID	Gene Symbol	Log$_2$FC	Description
**Oxidative stress genes**	15368	Hmox1	3.44	Heme oxygenase 1
	26570	Slc7a11	2.01	Solute carrier family 7 member 11
	14283	Fosl1	1.87	Fos-like antigen 1
	76650	Srxn1	1.41	Sulfiredoxin 1 homolog
	20856	Stc2	1.28	Stanniocalcin 2
	20856	Apoe	0.97	Apolipoprotein E
	11816	Etv5	0.90	Ets variant 5
	14629	Gclc	0.90	Glutamate-cysteine ligase, catalytic subunit
	50493	Txnrd1	0.88	Thioredoxin reductase 1
	11839	Areg	0.81	Amphiregulin
	12795	Plk3	0.81	Polo like kinase 3
	11911	Atf4	0.81	Activating transcription factor 4
	17250	Abcc1	0.70	ATP-binding cassette, sub-family C member 1
	230784	Sesn2	0.68	Sestrin 2
	14630	Gclm	0.64	Glutamate-cysteine ligase, modifier subunit
	72170	Chchd4	0.58	Coiled-coil-helix-coiled-coil-helix domain containing 4
	223646	Naprt	0.55	Nicotinate phosphoribosyltransferase
	20845	Star	0.55	Steroidogenic acute regulatory protein
	382985	Rrm2b	0.51	Ribonucleotide reductase M2 B
	19229	Ptk2b	-0.51	PTK2 protein tyrosine kinase 2 beta
	18854	Pml	-0.56	Promyelocytic leukemia
	13614	Edn1	-0.56	Endothelin 1
	211651	Fancd2	-0.57	Fanconi anemia, complementation group D2
	230738	Zc3h12a	-0.58	Zinc finger CCCH type containing 12A
	13605	Ect2	-0.62	Ect2 oncogene
	72774	Neil1	-0.69	Nei endonuclease VIII-like 1
	18595	Pdgfra	-0.79	Platelet derived growth factor receptor, alpha polypeptide
	20617	Snca	-0.86	Synuclein, alpha
	56338	Txnip	-0.97	Thioredoxin interacting protein
	12159	Bmp4	-1.04	Bone morphogenetic protein 4
	14281	Fos	-1.41	FBJ osteosarcoma oncogene
**Transcription factors**	14283	Fosl1	1.87	Fos-like antigen 1
	17869	Myc	1.14	Myelocytomatosis oncogene
	11910	Atf3	0.88	Activating transcription factor 3
	12608	Cebpb	0.78	CCAAT/enhancer binding protein, beta
	17121	Mxd3	-0.52	Max dimerization protein 3
	12615	Cenpa	-0.52	Centromere protein A
	12393	Runx2	-0.57	Runt related transcription factor 2
	320394	Cenpt	-0.61	Centromere protein T
	21417	Zeb1	-0.63	Zinc finger E-box binding homeobox 1
	14281	Fos	-1.41	FBJ osteosarcoma oncogene

Table S4. List of top 30 upregulated and downregulated proteins

1DIV vs Con				NEM/1D vs 1DIV			
Accession	Gene Symbol	Log_2 FC	up/down	Accession	Gene Symbol	Log_2 FC	up/down
Q9ET80	Jph1	2.52	up	Q9WUB3	Pygm	1.09	up
Q32M07	Ak8	2.05	up	Q7TQG1	Plekha6	0.90	up
P13412	Tnni2	1.61	up	Q9DCT8	Crip2	0.89	up
Q3LR78	Zbtb38	1.61	up	Q8BGH4	Reep1	0.75	up
P61208	Arl4c	1.52	up	Q6NZQ6	Znf740	0.64	up
P13541	Myh3	1.45	up	Q9CWF2	Tubb2b	0.63	up
Q3UX10	Tubal3	1.40	up	Q9CYC6	Dcp2	0.62	up
Q5XKE0	Mybpc2	1.37	up	A0A0B4J1F9	Tchh	0.61	up
Q64518	Atp2a3	1.37	up	O70250	Pgam2	0.56	up
Q8C0S1	Dis3l	1.35	up	P61514	Rpl37a	0.54	up
Q8CHT3	Ints5	1.34	up	Q6NZQ2	Ddx31	0.53	up
P20801	Tnnc2	1.30	up	Q8BP67	Rpl24	0.52	up
O09165	Casq1	1.29	up	Q9D3B1	Hacd2	0.50	up
Q9QZ47	Tnnt3	1.23	up	Q62186	Ssr4	0.50	up
E9PUL3	Clca3b	1.21	up	P97864	Casp7	0.49	up
Q8C7B8	Zswim4	1.18	up	P56379	Mp68	0.48	up
Q8CD54	Piezo2	1.16	up	Q3UUI3	Them4	0.47	up
Q62388	Atm	1.16	up	P68433	Hist1h3a	0.47	up
Q9CWF6	Bbs2	1.14	up	P70402	Mybph	0.46	up
Q8BX37	Papl	1.12	up	Q9DCD6	Gabarap	0.45	up
P97457	Mylpf	1.08	up	P07310	Ckm	0.45	up
Q14BI5	Myom2	1.08	up	G3X9G9	Mettl7a3	0.43	up
Q9JJH7	Trpm5	1.07	up	P62897	Cycs	0.43	up
P35762	Cd81	1.04	up	P56371	Rab4a	0.43	up
P07903	Ercc1	1.00	up	Q9CPQ8	Atp5l	0.42	up
Q62421	Sh3gl3	0.99	up	A2ADA5	Pusl1	0.40	up
P07934	Phkg1	0.99	up	B2RRE7	Otud4	0.40	up
P58771	Tpm1	0.99	up	Q99JW4	Lims1	0.39	up
Q5SX39	Myh4	0.97	up	Q8BY89	Slc44a2	0.39	up
Q91VB4	Hps3	0.97	up	Q9CRC0	Vkorc1	0.39	up
Q9CPQ8	Atp5l	-0.64	down	Q8BK08	Tmem11	-0.53	down
A2ABU4	Myom3	-0.65	down	Q01149	Col1a2	-0.54	down
Q9CQX6	Gm16286	-0.65	down	P13541	Myh3	-0.54	down
Q60872	Eif1a	-0.66	down	O88207	Col5a1	-0.54	down
G3X9G9	Mettl7a3	-0.67	down	Q91W97	Hkdc1	-0.55	down
Q9CRC0	Vkorc1	-0.67	down	Q61809	Lrrn1	-0.56	down
Q6NZQ2	Ddx31	-0.68	down	Q9Z148	Ehmt2	-0.56	down
Q9CR39	Wdr45b	-0.68	down	Q3UFS0	Zyg11b	-0.59	down
Q9CWS4	Cpsf3l	-0.69	down	Q9DBS2	Tprg1l	-0.60	down
Q62186	Ssr4	-0.70	down	P11087	Col1a1	-0.60	down
Q8BHJ6	Serinc5	-0.74	down	Q8BIQ6	Zfp947	-0.60	down
P62843	Rps15	-0.75	down	Q8BX37	Papl	-0.62	down
G3UW82	Myh2	-0.75	down	Q8R2N2	Cirh1a	-0.63	down
Q9WTN0	Ggps1	-0.77	down	Q8C1F4	Csgalnact2	-0.63	down
P83882	Rpl36a	-0.77	down	Q62388	Atm	-0.63	down
Q3UUI3	Them4	-0.78	down	P22907	Hmbs	-0.64	down
P68433	Hist1h3a	-0.79	down	Q91VB4	Hps3	-0.65	down
Q8BP67	Rpl24	-0.81	down	Q32M07	Ak8	-0.72	down
Q9CWF2	Tubb2b	-0.82	down	Q91X83	Mat1a	-0.72	down
P56379	Mp68	-0.87	down	Q8CE50	Snx30	-0.73	down
Q8HWE5	Mill2	-0.87	down	P04919	Slc4a1	-0.74	down
Q6NZQ6	Znf740	-0.99	down	Q8CD54	Piezo2	-0.75	down
Q8BGH4	Reep1	-1.07	down	Q62421	Sh3gl3	-0.78	down
Q9DCT8	Crip2	-1.08	down	Q9CWF6	Bbs2	-0.82	down
Q8CI43	Myl6b	-1.11	down	P61208	Arl4c	-0.84	down
P61514	Rpl37a	-1.15	down	Q3LR78	Zbtb38	-0.99	down
Q9JJW5	Myoz2	-1.33	down	E9PUL3	Clca3b	-1.01	down
P19123	Tnnc1	-3.33	down	Q8CHT3	Ints5	-1.08	down
P09542	Myl3	-3.60	down	Q3UX10	Tubal3	-1.52	down
P51667	Myl2	-3.99	down	Q9ET80	Jph1	-2.39	down

Table S5. List of top 30 upregulated and downregulated miRNAs

Accession	MiRNA name	Log_2FC	up/down	Accession	MiRNA name	Log_2FC	up/down
MIMAT0027797	mmu-miR-6948-3p	2.64	up	MIMAT0004877	mmu-miR-466c-5p	-2.42	down
MIMAT0022704	mmu-miR-489-5p	2.57	up	MIMAT0027820	mmu-miR-6960-5p	-2.06	down
MIMAT0049867	mmu-miR-12200-5p	2.55	up	MIMAT0017050	mmu-miR-32-3p	-1.91	down
MIMAT0003182	mmu-miR-494-3p	2.30	up	MIMAT0009416	mmu-miR-1949	-1.90	down
MIMAT0016989	mmu-miR-146a-3p	2.29	up	MIMAT0000246	mmu-miR-122-5p	-1.70	down
MIMAT0004704	mmu-miR-335-3p	2.29	up	MIMAT0000904	mmu-miR-215-5p	-1.70	down
MIMAT0004861	mmu-miR-877-5p	2.22	up	MIMAT0009396	mmu-miR-1933-5p	-1.70	down
MIMAT0019350	mmu-miR-3966	2.22	up	MIMAT0014834	mmu-miR-3064-5p	-1.70	down
MIMAT0017055	mmu-miR-219a-1-3p	2.22	up	MIMAT0025113	mmu-miR-6369	-1.70	down
MIMAT0027699	mmu-miR-6899-3p	2.22	up	MIMAT0017084	mmu-miR-181b-2-3p	-1.56	down
MIMAT0020629	mmu-miR-5121	2.01	up	MIMAT0003486	mmu-miR-491-5p	-1.45	down
MIMAT0000766	mmu-miR-335-5p	1.87	up	MIMAT0017258	mmu-miR-500-5p	-1.45	down
MIMAT0004875	mmu-miR-466b-5p	1.87	up	MIMAT0003508	mmu-miR-501-5p	-1.45	down
MIMAT0027822	mmu-miR-6961-5p	1.87	up	MIMAT0017283	mmu-miR-598-5p	-1.45	down
MIMAT0027938	mmu-miR-7017-5p	1.87	up	MIMAT0009390	mmu-miR-1927	-1.45	down
MIMAT0035718	mmu-miR-935	1.87	up	MIMAT0009394	mmu-miR-1931	-1.45	down
MIMAT0017020	mmu-miR-26a-1-3p	1.87	up	MIMAT0009398	mmu-miR-1934-5p	-1.45	down
MIMAT0027765	mmu-miR-6932-3p	1.87	up	MIMAT0017182	mmu-miR-450a-1-3p	-1.45	down
MIMAT0028091	mmu-miR-7092-3p	1.87	up	MIMAT0003898	mmu-miR-760-3p	-1.45	down
MIMAT0020633	mmu-miR-5123	1.82	up	MIMAT0027805	mmu-miR-6952-3p	-1.45	down
MIMAT0028088	mmu-miR-7091-5p	1.82	up	MIMAT0027831	mmu-miR-6965-3p	-1.45	down
MIMAT0031426	mmu-miR-8120	1.82	up	MIMAT0027891	mmu-miR-6994-3p	-1.45	down
MIMAT0000519	mmu-miR-200a-3p	1.82	up	MIMAT0029909	mmu-miR-7689-3p	-1.45	down
MIMAT0002107	mmu-miR-466a-3p	1.82	up	MIMAT0009413	mmu-miR-1947-5p	-1.45	down
MIMAT0031397	mmu-miR-1291	1.70	up	MIMAT0010560	mmu-miR-1249-3p	-1.40	down
MIMAT0005293	mmu-miR-467e-5p	1.67	up	MIMAT0000209	mmu-miR-129-5p	-1.37	down
MIMAT0022353	mmu-miR-3544-5p	1.67	up	MIMAT0009393	mmu-miR-1930-5p	-1.37	down
MIMAT0014905	mmu-miR-3092-5p	1.62	up	MIMAT0000216	mmu-miR-187-3p	-1.37	down
MIMAT0005837	mmu-miR-1187	1.58	up	MIMAT0001342	mmu-miR-425-3p	-1.37	down
MIMAT0029894	mmu-miR-181b-1-3p	1.42	up	MIMAT0017067	mmu-miR-181b-1-3p	-1.26	down
